# *Ganoderma lucidum:* Insight into antimicrobial and antioxidant properties with development of secondary metabolites

**DOI:** 10.1016/j.heliyon.2024.e25607

**Published:** 2024-02-04

**Authors:** Md Faruque Ahmad, Abdulrahman A. Alsayegh, Fakhruddin Ali Ahmad, Md Sayeed Akhtar, Sirajudeen S. Alavudeen, Farkad Bantun, Shadma Wahab, Awais Ahmed, M. Ali, Ehab Y. Elbendary, António Raposo, Nahla Kambal, Mohamed H. Abdelrahman

**Affiliations:** a Department of Clinical Nutrition, College of Applied Medical Science, Jazan University, Jazan, 45142, Saudi Arabia; b Department of Basic and Applied Science, School of Engineering and Science, G.D Goenka University, Gru Gram, 122103, Haryana, India; c Department of Clinical Pharmacy, College of Pharmacy, King Khalid University, AlFara, Abha, 62223, Saudi Arabia; d Department of Microbiology and Parasitology, Faculty of Medicine, Umm Al-Qura University, Makkah, Saudi Arabia; e Department of Pharmacognosy, College of Pharmacy, King Khalid University, Abha, 62529, Saudi Arabia; f Department of Management, Shri JJT University, Rajasthan, Post code; 333010, India; g Department of Pharmacognosy, CBS College of Pharmacy & Technology (Pt. B. D. Sharma University of Health Sciences), Chandpur, Faridabad, Haryana, 121101, India; h CBIOS (Research Center for Biosciences and Health Technologies), Universidade Lusófona de Humanidades Tecnologias, Campo Grande 376, 1749-024, Lisboa, Portugal; i College of Applied Medical Sciences, Medical Laboratory Sciences, Jazan University, Jazan, 45142, Saudi Arabia

**Keywords:** Antimicrobial, Antioxidant, Secondary metabolites, Triterpenoids and polysaccharides

## Abstract

*Ganoderma lucidum* is a versatile mushroom. Polysaccharides and triterpenoids are the major bioactive compounds and have been used as traditional medicinal mushrooms since ancient times. They are currently used as nutraceuticals and functional foods. *G. lucidum* extracts and their bioactive compounds have been used as an alternative to antioxidants and antimicrobial agents. Secondary metabolites with many medicinal properties make it a possible substitute that could be applied as immunomodulatory, anticancer, antimicrobial, anti-oxidant, anti-inflammatory, and anti-diabetic. The miraculous properties of secondary metabolites fascinate researchers for their development and production. Recent studies have paid close attention to the different physical, genetic, biochemical, and nutritional parameters that potentiate the production of secondary metabolites. This review is an effort to collect biologically active constituents from *G. lucidum* that reveal potential actions against diseases with the latest improvement in a novel technique to get maximum production of secondary metabolites. Studies are going ahead to determine the efficacy of numerous compounds and assess the valuable properties achieved by *G. lucidum* in favor of antimicrobial and antioxidant outcomes.

## 1 Introduction

**Fig. 1:**
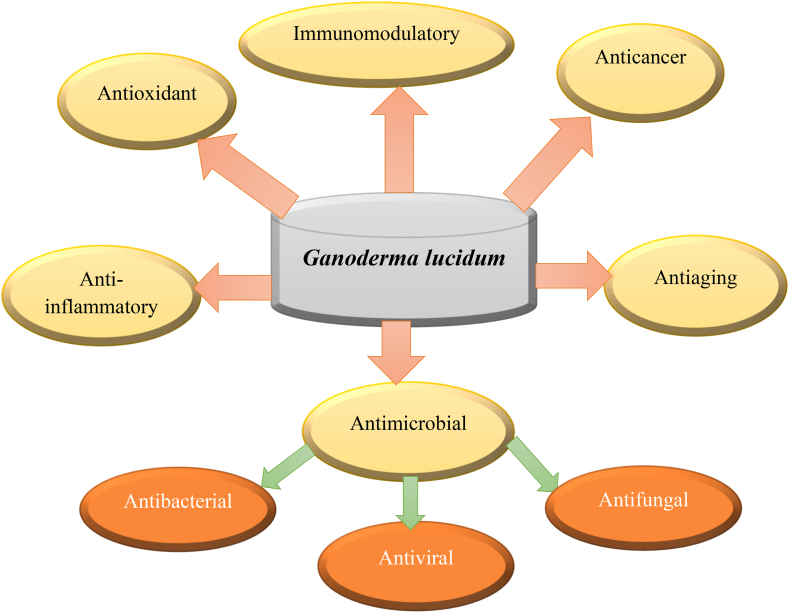
Potential diverse action of *G. lucidum* as a health promoting agent.

*G. lucidum* is one of the most common medicinal mushrooms that have been used globally. It has been applied as a traditional Chinese medicine to promote health. It has a long history of consumption for endorsing health and longevity in Japan, China, India, and other Asian nations. It is known by various synonyms, including reishi, lingzhi, and mushroom of immortality, across the world. *G. lucidum* active constituents and their potency are listed in the Chinese Pharmacopoeia, Therapeutic Compendium, and American Herbal Pharmacopoeia [1,2]. *G. lucidum* grows in various habitats around the world. Some of its common habitats include deciduous forests. *G. lucidum* is often found growing on dead or dying trees in deciduous forests; these trees include oak, maple, and elm. In coniferous forests, mushrooms can also be found growing on coniferous trees such as pine, spruce, and fir. In temperate regions, *G. lucidum* can be found growing on a variety of trees, including birch, beech, and poplar. Overall, *G. lucidum* is a versatile fungus that can thrive in a variety of habitats around the world [[3], [4], [5], [6]]. Taxonomic studies have described over 300 species in the genus *Ganoderma*, and most of them are spread in tropical regions [7]. Above 430 secondary metabolites, over 380 terpenoids such as ganoderic acids (GAs), lucidenic acids, aldehydes, esters, alcohols, lactones, glycosides, and meroterpenoids were isolated from Ganoderma [[8], [9], [10]]. Terpenoids and steroids from *Ganoderma* showed significant biological activity. So far, above 240 secondary metabolites have been obtained only from *G. lucidum* species [11]. Polysaccharides and triterpenoids are the main biologically active constituents that make *G. lucidum* a potential agent [12]. Triterpene compounds are obtained from lanosterol and include ganoderic acids, lucinedic acids, ganodermic acids, lucidones, and ganodermic alcohols. While more than 200 polysaccharides, like α-D-glucans, β-glucans, β-D-glucans and polysaccharide-protein complexes, have been found in fruiting bodies, mycelia, and spores [[13], [14], [15], [16]]. Other complex compounds include pro-vitamin D2, alkaloid, glycoproteins, nucleotides, coumarins, lysozyme, flavonoids, enzymes, long-chain fatty acids, essential amino acids, phenols, sterols, germanium, and different minerals like copper, zinc, selenium, potassium, calcium, phosphorus, magnesium, and iron, as reported in various research studies [[17], [18], [19], [20], [21], [22]]. Leucine and lysine are found in very large amounts in *G. lucidum*, and it also has a lot of polyunsaturated fatty acids compared to the total number of fatty acids, making it a potential agent for our health [20,23]. There are more than 100 products on the market that contain reishi [24]. *G. lucidum* efficacy has been proven in a wide range of ailments that include anticancer [25,26], antioxidant [27], antidiabetic [28], antihyperlipidemic [29,30], antimutagenic [31], anti-aging [32], antimicrobial (antiviral, antibacterial and antifungal) [33,34], hepatoprotective [35,36], anti-hyperpigmentation [1], cardioprotective [37], pro-apoptotic [38], anti-androgenic [39], anti-allergic [40], antinociceptive [41] and improve physical frailty [42]. Review articles on *G. lucidum* antimicrobial and antioxidant properties are written to deliver a comprehensive analysis of the current scientific literature related to the antimicrobial and antioxidant effects of *G. lucidum* and evaluate the available evidence to determine the potential properties and its recent advances in the development of secondary metabolites. The diverse action of *G. lucidum* is depicted in Fig. 1.

## 2 Materials and methods

The present comprehensive review collected evidence through diverse databases that include PubMed, Google Scholar, the Saudi Digital Library, and the Cochrane Library until April 2023. Keywords used: *G. lucidum*, *G. lucidum* biological active constituents, *G. lucidum* triterpenoids extracts, *G. lucidum* polysaccharides extracts, secondary metabolites, ganoderic acids. Phrases that have been used include “*G. lucidum* antimicrobial properties'’, “antibacterial effects of *G. lucidum*’’, “antifungal effects of *G. lucidum*’’, “antiviral effects of *G. lucidum*’’, antioxidant potency of *G. lucidum*," "antioxidant efficacy of *G. lucidum*, “development of secondary metabolites, factors influencing the production of secondary metabolites, “effects of nutrients in the production of secondary metabolites,’’ “Effects of biochemicals in production of secondary metabolites'’ and “future prospects of *G. lucidum* as a antioxidants and antimicrobial agent. English-language published articles were chosen to find antioxidants and antimicrobial effects of *G. lucidum* in the literature survey. Literature studies were collected from the last 39 years of published research data, from 1984 to 2023.

## 3 Antimicrobial activity

**Fig. 2:**
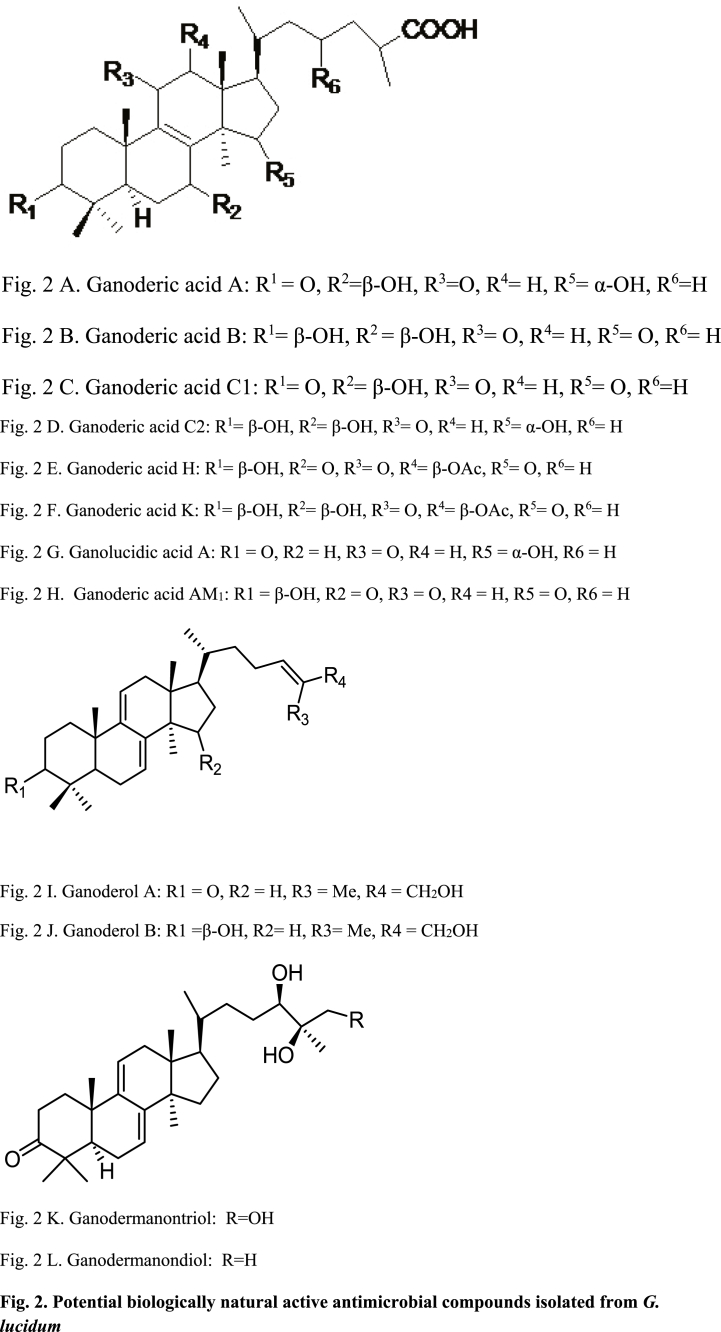
(A). Ganoderic acid A: R^1^=O, R^2^ = β-OH, R^3^=O, R^4^=H, R^5^ = α-OH, R^6^=H (B). Ganoderic acid B: R^1^ = β-OH, R^2^ = β-OH, R^3^=O, R^4^=H, R^5^=O, R^6^= H (C). Ganoderic acid C1: R^1^=O, R^2^ = β-OH, R^3^=O, R^4^=H, R^5^=O, R^6^=H (D). Ganoderic acid C2: R^1^ = β-OH, R^2^ = β-OH, R^3^=O, R^4^=H, R^5^ = α-OH, R^6^= H (E). Ganoderic acid H: R^1^ = β-OH, R^2^=O, R^3^=O, R^4^ = β-OAc, R^5^=O, R^6^= H (F). Ganoderic acid K: R^1^ = β-OH, R^2^ = β-OH, R^3^=O, R^4^ = β-OAc, R^5^=O, R^6^= H (G). Ganolucidic acid A: R1=O, R2=H, R3=O, R4=H, R5 = α-OH, R6 = H (H). Ganoderic acid AM_1_: R1 = β-OH, R2=O, R3=O, R4=H, R5=O, R6 = H (I). Ganoderol A: R1=O, R2=H, R3 = Me, R4=CH_2_OH (J). Ganoderol B: R1 = β-OH, R2=H, R3 = Me, R4=CH_2_OH (K). Ganodermanontriol: R=OH (L). Ganodermanondiol: R=H Potential biologically natural active antimicrobial compounds isolated from *G. lucidum*.

Microorganisms like bacteria, viruses, fungi, and protozoa have always been a danger to health. Bioactive compounds and mushroom extracts have shown promise in the search for new antimicrobial agents. Even though there are numerous synthetic antimicrobial agents available to treat infectious diseases, drug resistance and toxicity are still challenging issues, particularly when used for a long period of time. In concern for lesser side effects and safety, most people are heading towards herbal drugs, nutraceuticals, and food supplements [43]. So, it is the need of the hour to search for new natural antimicrobial agents and alternative medicines that act as an alternative to current medicines [44,45]. The goal of natural antimicrobial agents is mainly to treat the pathogens, stop their growth without harming normal cells, and protect from microbial resistance [33,46]. Different antimicrobial agents derived from natural sources are being researched. The mechanisms of *G. lucidum* against microorganisms are still not well described. Even though the extracts have a number of biologically active constituents, such as glycosides, carbohydrates, triterpenoids, tannins, and phenolic compounds that have some antimicrobial activity, most of them act in extract form. Some of the biologically active constituents that have specific antiviral properties include ganoderic acid (GA)-A, GA-B, GA-T, GA-Q, GA-C1, GA-C2, GA-H, GA-DM, ganoderol A, ganoderol B, ganodermanondiol, and ganodermanontriol (Fig. 2) [[47], [48], [49]]. While protein ganodermin and organic and aqueous extract of *G. lucidum* exhibit antifungal properties [50,51]. Furthermore, Ergosta-5,7,22-trien-3β-yl acetate, ergosta-7,22-dien-3β-yl acetate, ergosta-7,22-dien-3β-ol, ergosta-5,7,22-trien-3β-ol, ganodermadiol, mycelia and fruiting body protein extracts and polysaccharides of *G. lucidum* exhibited antibacterial activity [52]. *G. lucidum* extracts have a wide range of antimicrobial activities; they act against both gram-positive and gram-negative bacteria. Most of the research has been done on mycelium and fruiting body extracts, with only a few studies conducted on polysaccharides and other secondary metabolites that are the core active constituents of *G. lucidum.* In general, *G. lucidum* aqueous and organic solvent (methanol, hexane, ethyl acetate, and dichloromethane) extracts are commonly applied against *Bacillus cereus (B. cereus)*, *Staphylococcus aureus* (S. *aureus*), *Enterobacter aerogenes*, *Pseudomonas aeruginosa (P. aeruginosa),* and *Escherichia coli* (*E. coli)* [45,48,53].

**Fig. 3:**
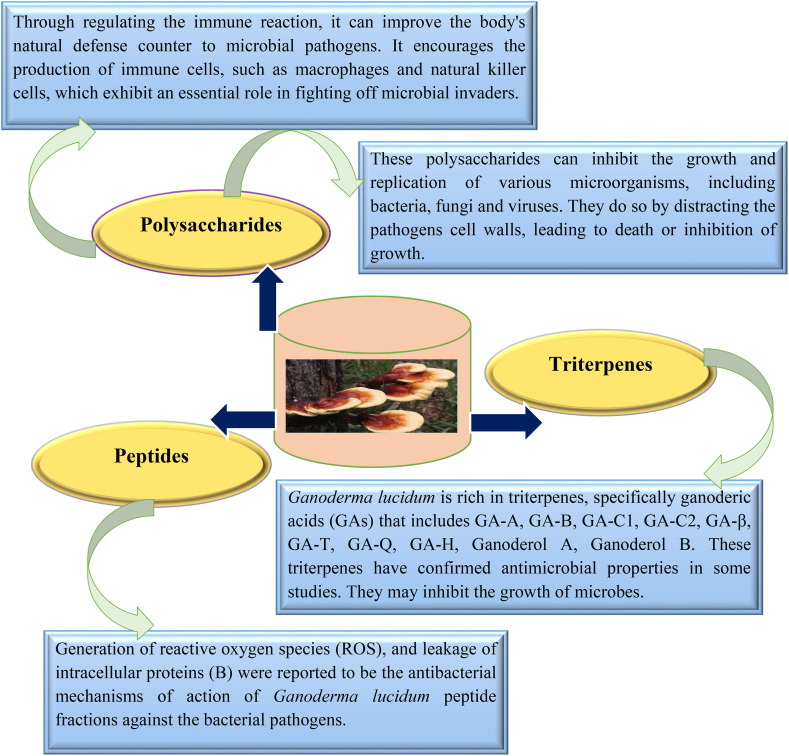
Potential proposed antimicrobial mechanisms of action exhibited by *G. lucidum* isolated compounds.

The antimicrobial mechanisms of *G. lucidum* are not yet entirely revealed and require further research. However, numerous potential mechanisms have been proposed based on the existing studies. It's significant to note that these mechanisms may show a discrepancy based on the specific bioactive compounds present in *G. lucidum* extracts [48,[54], [55], [56]]. Proposed antimicrobial mechanisms can be seen in Fig. 3.

### 3.1 Antibacterial effects

Antibacterial substances are medicines that are used to treat bacterial diseases. There are a variety of medicines available [57]. According to a new report, antibacterial use has increased by 46 % in humans [58]. There are numerous bacteria that can cause diseases in humans. The spectrum of disease ranges from pandemics like cholera and plague to common skin infections caused by *Streptococcus* [58,59]. As the world is advancing and there is more equipment, knowledge, and expertise available, it still remains a challenge to find out and treat bacterial infections properly [60]. Lower respiratory tract infections again remain the fourth leading cause of death. In elderly people, especially in the lower respiratory tract, infections, whether viral or bacterial, remain one of the toughest clinical conditions to treat [61]. There are multiple antibiotics available to treat these infections. It becomes difficult when resistance develops and the infection becomes untreatable. So, it is the need of the hour to find such natural compounds that could act against such pathogens without resistance or toxicity [48,[62], [63], [64], [65]].

It has been reported in various studies that compounds extracted from *G. lucidum* have potent antibacterial activity [66]. Different types of extracts like aqueous, hexane, chloroform, methanol, and ethanol from the fruiting body and mycelia of *G. lucidum* exhibit potential action against *E. coli, P. aeruginosa, S. aureus,* and *Staphylococcus pyogenes (S. pyogenes*) [67,68]. The research mentions that these bacterial specimens were multidrug-resistant, and *G. lucidum* extracts showed activity and areas of inhibition against them [69]. Ethanol extracts have been shown to have antibacterial action against *S. aureus* in another study carried out in Jakarta. Another more research explains that the ethanol extract of *G. lucidum* has activity against *S. aureus* in concentrations of 900 and 600 mg/ml [70]. Confirmed antibacterial activity against various bacteria like *Bacillus subtilis, E. coli, Acinetobacter, S. aureus, Pseudomonas, Acetobacter, Brevibacillus brevis, Salmonella typhi, Rhizobium* for *Vigna mung*, and *Rhizobium* for *Cicer arietinu* [71]. Similar results have been found in different laboratories in Namibia and South India [72,73]. In Namibia, like in China, *G. lucidum* has been used for many years as a traditional medicine for treating various skin and wound infections. The authors validated the effects of benzene extract, and they found definitive antibacterial activity of the extract on colonies of *E. coli* and *Neisseria meningitides* [73]. Furthermore, in an antibacterial study, it was reported that different concentrations (0.5 mg/100 μl and 1.0 mg/100 μl) of *G. lucidum* extracts exhibited different inhibitory effects, with the methanol and aqueous extracts (0.5 mg/100 μl) showing noticeable results in comparison to other extracts. Methanol extracts of *G. lucidum* possessed strong antimicrobial action against *Proteus vulgaris*, narrowly followed by *Enterococcus faecalis*. It revealed moderate results against *Salmonella typhimurium, P. aeruginosa, and Listeria monocytogenes* at the same concentration. But it was highly reduced in cases of *Streptococcus mutans, B. subtilis,* and *Klebsiella pneumonia.* The antibacterial activity of the aqueous extract exhibited less than that of the methanol extract. Since most active components are often water-insoluble and methanol is a superior extracting solvent than water, it is believed that low-polarity solvents made from organic compounds will provide a more active extract [74,75]. The terpenes, polysaccharides, and lectins found in *G. lucidum* fruit bodies, as well as their solubility in the extracts employed for the current study, were evaluated to assess their antibacterial properties [45,75]. The minimum inhibitory concentration (μg/ml) of different bacteria in different extracts can be seen in Table 1.

**Table 1:** Minimum inhibitory concentration (μg/ml) of methanol and water extracts of *G. lucidum* against different bacteria.

**Bacteria**	**Minimum Inhibitory Concentration (MIC)**	**References**	
	Water (μg/ml)	Methanol (μg/ml)	
***B. subtilis***	31.25	31.25	[45]
***P. vulgaris***	31.25	31.25	[45]
***Streptococcus*** ***Mutans***	62.50	62.50	[45]
***Klebsiella pneumoniae***	31.25	31.25	[45]
***Salmonella typhimurium***	31.25	31.25	[45]
***L. monocytogenes***	31.25	–	[45]
***P. aeruginosa***	31.25	31.25	[45]
***Enterococcus faecalis***	31.25	31.25	[45]

Similarly, one more experimental study in Jabalpur, India, developed various solvent extracts from complete mushroom powder. They found the acetone extract of *G. lucidum* was the most active against six species, namely *E. coli*, *S. aureus*, *K. pneumoniae*, *B. subtilis*, *S. typhi*, and *P. aeruginosa*, and it was concluded that the antibacterial activity of the extract was most potent against *K. pneumonia* [76]. Antibacterial activity against *Corynebacterium diphtheriae* has also been evaluated. *C. diphtheriae* causes one of the most fatal diseases called diphtheria; if timely antibiotics and antibody serum are not administered, it turns deadly. In a study conducted in Mumbay, it was found that *G. lucidum* extracts were discovered to have bactericidal properties. They prepared an aqueous extract, an acetone extract, a methanol extract, and a chloroform extract of *G. lucidum*. They found a definite zone of inhibition on the culture plate while using these extracts. The most susceptible bacteria found were *C. diphtheriae*, and the least activity was found against *Pseudomonas* [67]. The antibacterial activity of *Ganoderma* extract extends to plant pathogens as well. In Mexico, researchers identified a polysaccharide in the extract of *G. lucidum* that was effective against phytopathogens [63]. *G. lucidum* has been explored as a potential source for the synthesis of silver nanoparticles (AgNPs). Recent research by Constatntin et al. (2023) reported that mycelia from *G. lucidum* have aqueous extracts that include bioactive substances that could be used to create nanoparticles with antibacterial properties. AgNPs produced by *G. lucidum* during biosynthesis mostly have antimicrobial effects on a variety of bacterial species, including *E. coli, P. aeruginosa, and S. aureus* [66]. After considering all these published studies in reputed journals, it has been concluded that *G. lucidum* has potential antibacterial properties, but isolation and characterization of the active ingredients are needed. Further evaluation and drug trials have been needed to bring these extracts to the market as medicines. The antibacterial effects of *G. lucidum* reported by various experimental studies in different countries are explored in Table 2.

**Table: 2:** Antibacterial effects of *G. lucidum* reported by various experimental studies in different countries using different solvent extracts.

Type of extract	Country	Preparation	Activity against	MIC	References
Fruit body extract	Indonesia	Ethanol extract	*E. coli* and *S. aureus*		[70]
Extracted from Complete mushroom	India	Ethanol extract	Multi drug resistant strains of *E. coli, S. typhimurium, P. aerugniosa, S. aureus* and *strep pyogenes*		[68,69]
Complete extract	Turkey	Dichloromethane extract	*S. aureus, E. coli* and *P. aeruginosa*	200 μg/ml	[77,78]
Extracted from Complete mushroom	Bangladesh	Aqeous extract	*B. subtilis*, *E. coli*, *S. aureus*, *Pseudomonas*, *Acinetabactor*, *Bravibacillus bravis*, *S. typhi*, and *Rhizobium* for *Cicer arietinum*		[79,80]
Crude extract- complete mushroom	Namibia	Benzene extract	*E. coli* and *Neisseria Meningitidis*		[73,81]
Complete extract	Turkey	Methanol extract	*E. faecalis, A. baumannii, C. albicans, C. glabrata* and *C. krusei*	100 μg/ml	[77]
Complete extract	Turkey	Methanol extract	*S. aureus, E. coli* and *P. aeruginosa*	200 μg/ml	[77]
Complete mushroom or mycelia	India	Methanol, acetone, chloroform and aqeous extracts	*S. aureus, B. subtilis, Corynebacterium diphtheriae,* *E. coli, K. pneumoniae, S. typhi, Pseudomonas* and *Proteus mirabilis*		[67,82]
Fruiting body	India	Peptide fractions	*S. aureus, E. coli* and *S. typhi*		[54,83,84]
Extracted from complete mushroom	India	Methanol extract	*E. coli, S. typhimurium* and *B. subtilis*	1 mg/well	[72,85]
Extracted from complete mushroom	India	Methanol extract	*P. rimosus* and *N. floccossa*	500 μg/well	[72]
Not mentioned	Mexico	Methanol and aqueous extracts	*Agrobacterium rhizogenes, Acidovorax avenae, Agrobacterium tumefaciens, Burkholdia cepacian, Brenneria quercina, P. fluorescens, Rathayibacter tritici, P. syringae* and *Xanthomonas campestris*		[63,86]
*Ganoderma* mycelial extracts of different species	Mexico	Chloroform and methanol extracts	*Clavibacter michiganensis*	31.5–1000 mg/ml	[86]
Fruiting bodies	India	Methanol and ethyl acetate	*S. aureus* and *Enterobacter aerogenes*	2.5 mg/ml	[84,87,88]
Solvent extract- Extracted from complete mushroom	India	Acetone extract	*E. coli, S. aureus, K. pneumonia, B. subtilis, Salmonella typhae* and *P. Aeruginosa*		[67,76]
Mycelia protein and fruiting body	Thailand	Protein extract	*B. subtilis, B. cereus, Staphylococcus epidermidis, S. aureus, E. coli* and *P. aeruginosa*		[89,90]
Fruiting bodies	Iran	Hexane and chloroform extract	*S. aureus, B. subtilis, P. aeruginosa* and *E. coli*		[91,92]
Fruiting bodies	India	Methanol extracts	*B. cereus* and *E. coli*	1.25 mg/ml	[88]
Fruiting bodies	India	Aqueous, hexane, dichloromethane, ethyl acetate and methanol extracts.	*B. subtilis, E. faecalis, Listeria moncytogenes, Streptococcus mutans, P. vulgaris, Salmonella typhimurium, K. pneumoniae* and *P. aeruginosa*		[45,93]
*G. lucidum* mycelium extract	UK	Ethanol extract	*Shigella sonnei, Salmonella enteritidis, Listeria monocytogenes, Pseudomonas aeruginosa* and *E. coli*	3 mg/ml	[94]
*G. lucidum* mycelium extract	UK	Methanol extract	*S. aureus*	2 mg/ml	[94]

### 3.2 Antiviral effects

The goal of antiviral drugs is to find antiviral agents that can prevent viruses from spreading without harming normal cells. Now is the time to look for natural agents that might be able to kill viruses without making them resistant or having other side effects [95,96]. Several experimental studies have shown that *G. lucidum* could be a safe alternative to antiviral drugs [97]. Mushrooms are an enormous source of bioactive metabolites with little or negligible toxicity. Nevertheless, developing antiviral medications or vaccines for the viral infection is a challenging task, and currently, a natural source of therapy is a source to improve the immune system and reduce the death rate [98]. *G. lucidum* bioactive components exhibit a dynamic role in numerous human ailments, and they are measured as a source of current medication [44].

Millions of people around the world have the human immunodeficiency virus (HIV). HIV has a lot of different genes and comes mainly in two categories: HIV-1 and HIV-2, with numerous subtypes [99]. The HIV virus leads to AIDS by weakening the T lymphocytes, which are the body's defense cells, and weakening the immune system. T cell presence is needed for an immune response that plays a key role in various types of infections. HIV management strategies currently delay AIDS progress [100,101]. Protease inhibitors play an important role by binding selectively to viral proteases and stopping protein precursor proteolytic cleavage that is needed for infectious viral particles [[102], [103], [104]]. But the long-term effects of these drugs are greatly hampered by the development of strains that are resistant to drugs and/or toxic. Recent research has shown that many natural substances can be used to fight HIV [105]. In the search for and development of antiviral drugs, preventing viral protease is an elementary goal. Various antiviral triterpenoids of *G. lucidum* exhibit antiviral properties by acting on HIV-1 protease, like ganolucidic acid A, 3-5-dihydroxy-6-methoxyergosta-7,22-diene, GA-A, GA-B, ganodermanondiol, ganodermanontriol, and lucidumol B [49,106,107]. Twenty-five metabolites were obtained from *G. sinnense* fruiting bodies, and it has been reported that ganoderic acid GS-2, 20(21)-dehydrolucidenic acid N, ganoderiol F, and 20-hydroxylucidenic acid N had the potential to stop HIV-1 protease action [108]. Also, lucidenic lactone and lucidenic acid O found in the fruiting bodies of *G. lucidum* stop DNA polymerase-α, DNA polymerase-β, and HIV-1 RT activity [106,109,110].

It has been observed in a study that *Ganoderma* was tested in an *in vitro* cell culture model to see its activity against HIV-1. The results showed that *Ganoderma* stopped HIV-1 replication and cut the production of primary and secondary virus transcriptions [111]. It has also been found in an experiment that *G. lucidum* extracts also inhibit HIV-1 reverse transcriptase activity and that triterpenoids in the fungus have a lot of potential for treating HIV [112]. It has also been found in an experiment that *G. lucidum* extracts also inhibit HIV-1 reverse transcriptase activity and that triterpenoids in the fungus have a lot of potential for treating HIV [112]. *Ganoderma* was found to be effective against enterovirus 71 (EV-71) and influenza virus (flu) in addition to HIV [97,[112], [113], [114]]. Among enterovirus subtypes, EV-71 is the most probable reason for severe neurological diseases in children under 6 years of age. Antiviral drugs used to treat EV-71 only ease symptoms and don't stop the virus from spreading [97]. Triterpenoids from *G. lucidum* named ganoderic acid Y (GLTB) and lanosta-7,9(11),24-trien-3-one,15; 26-dihydroxy (GLTA) inhibit human rhabdomyosarcoma cells from viruses. Similarly, the results of molecular docking computations revealed that the uncoating process of the virus was stopped by GLTA and GLTB binding from capsid proteins at a hydrophobic pocket (F site), which stops EV-71 from replicating [115]. This experiment showed that GLTB and GLTA from *G. lucidum* could be used as drugs to protect against the EV-71 virus. Neuraminidase (NA) is a key factor pivotal in letting flu out of host cells. Owing to this, NA inhibitors have received a lot of attention in influenza treatment. Zhu et al. (2015) conducted an *in vitro* NA inhibition assay to study the effects of 31 *G. lucidum* triterpenoids. They found that GA-TQ and GA-TR could be used to stop the spread of H1N1 and H5N1 viruses [113]. It has also been seen in silico docking results that showed that GA-TQ and GA-TR blocked H5N1 and H1N1 NA activity by interacting with amino acid residues Arg292 or Glu119 of NA.

Traditional Chinese herbal medicine (TCM) was used a lot during the pandemic to treat COVID-19. This got the attention of people all over the world. TCM has always used groups of herbs that can be thought of as a mix of different active ingredients [116,117]. So, the binding of active components to different targets can affect different signal pathways and create synergistic effects that include treating viral respiratory infections [[118], [119], [120]]. Al-Jumaili et al. (2020) reported that the addition of *G. lucidum* to the treatment of COVID-19 increased the patient's lymphocyte counts. Studies, particularly against COVID-19, have not been conducted more, but it is expected that it will be a potent agent against coronaviruses due to its dual effects of immunomodulatory and antiviral activity [121]. *G. lucidum* immunomodulatory effects have become useful tools for treating the diseases that come with viral infections by activating macrophages, T lymphocytes, NK cells, and cytokines [121,122]. *G. lucidum* immunomodulatory mechanisms stimulate both innate and adaptive immune responses. *Ganoderma* glucans boost the signals sent by pattern recognition receptors (PRRs), which leads to protective inflammatory responses that stop pathogen-associated infections. Immunomodulators from *G. lucidum* are under study to find out their various modes of action and their efficacy in developing antiviral drugs that could be effective in COVID-19 [[122], [123], [124]]. Different species of *Ganoderma* and their effects against various viral infections can be seen in Table 3.

**Table 3:** Antiviral potency of *Ganoderma* various species against different viruses and their effects.

*Ganoderma* species	Compounds	Viruses	Effects	References
*Ganoderma lucidum*	Hesperetin and ganosin B	Dengue virus	Prevent DENV2 NS2B-NS3 Protease	[125]
	(GLTA) and Ganoderic acid Y	Enterovirus 71	Prevent EV-71 replication and block the virus adsorption to the cells	[97]
	Ganoderic acid A, B, C1, H and β	Human immunodeficiency virus (HIV)	Reveal potential effects against HIV infection and prevent HIV protease enzyme	[112,126]
	Proteoglycan	HSV-1 and HSV-2	Pre- and co-treatment effects	[127,128]
	Ganoderic acid H	Hepatitis	Inhibition of the production of HBV surface antigen	[129]
	Ganoderiol-F and Ganodermanontriol	HIV-1 protease	Reveal potential effects against HIV infection	[106]
*Ganoderma pfeifferi*	Ganodermadiol	HSV-1	Protection of cells	[130]
	ganoderone A and lucialdehyde B	HSV-1	Pre-treatment effect	[130]
	Ganodermadiol and lucidadiol	Influenza virus type A and HSV type 1	Protection of cells	[130]
	Ganoderone-C and lucialdehyde B	Influenza virus type A	inhibition of the growth of influenza virus	[131]
*G. sinense*	Ganoderic acid GS-2,20-hydroxylucidenic acid N, 20 dehydrolucidenic acid N and ganoderiol F	HIV-1 protease	Reveal potential effects against HIV infection	[108]
*G. colossum*	Colossolactone V, colossolactone VII, colossolactone VIII, colossolactone A and schisanlactone- A	HIV 1	Anti-HIV-1 Protease Activity	[132]
	Ganomycin-I and Ganomycin B	HIV 1	Anti-HIV-1 Protease Activity	[132]

### 3.3 Antifungal effects

Fungi exhibit a remarkable role in the production of significant antibiotics, the best-known of which is penicillin. Nevertheless, the study of the development and production of antibiotics through mushrooms has not been more widely reported [133]. Mushrooms have been assumed to have weak antifungal activity. Very recently, mushrooms have become of interest due to the occurrence of secondary metabolites, which possess a wide range of antimicrobial activities [44,134]. The protein ganodermin (15-kDa) was detected and analysed as an antifungal protein for the first time ever in the fruiting body of *G. lucidum*. Mycelial growth of *Fusarium oxysporum, Botrytis cinerea*, and *Physalospora piricola* was reported to be inhibited by ganodermin at varying degrees of IC values [135]. Literature surveys reveal the antifungal potential reports of *G. lucidum* to a very limited extent, but they are the most promising ones to suit the significant purpose of cure. One more study was reported to indicate the antifungal activity of *G. lucidum* extract against *Trichoderma viride*, which was found to be very significant upon comparing it with the established standard, i.e., ketoconazole and bifonazole [136]. In other research, organic and aqueous extracts of *G. lucidum* were found to be potential agents against plant pathogenic fungi, including *Fusarium oxysporum* and *Alternaria alternate* [137].

**Fig. 4:**
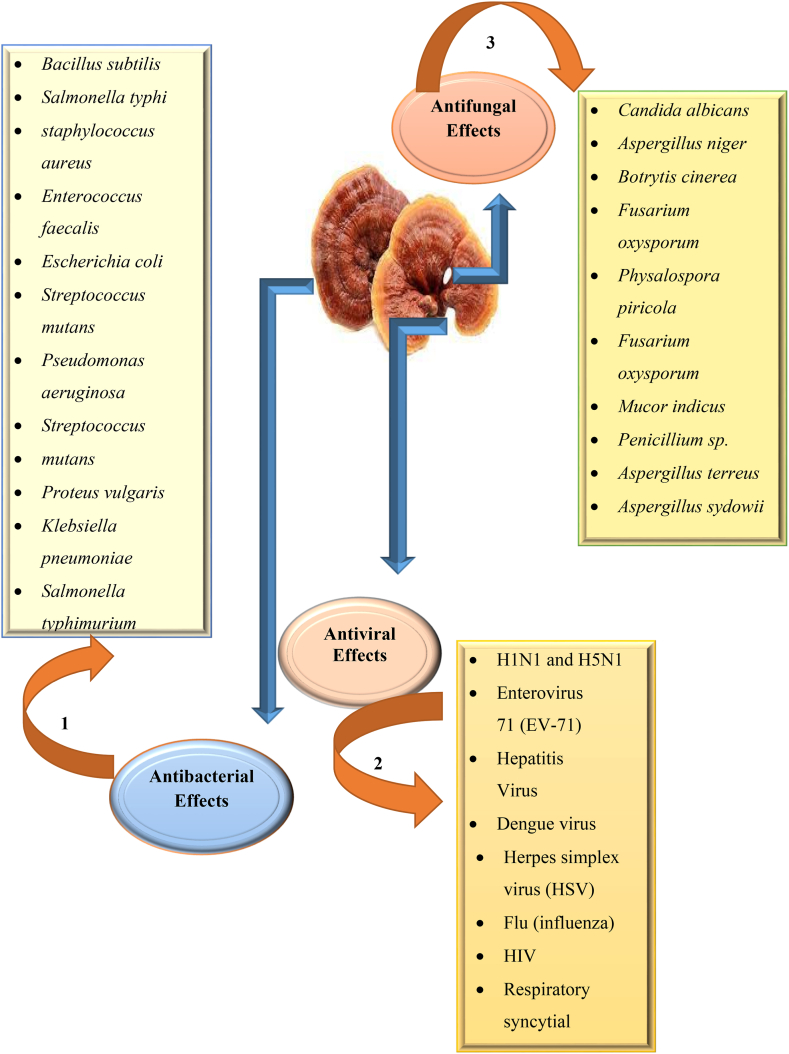
***G. lucidum* potential antimicrobial properties against bacteria, viruses, and fungi with biologically active constituents affect these microorganisms. *Abbreviation:*** Numeric forms (1, 2, and 3) express the different sources of biological active constituents that produce antimicrobial properties. 1: ethanol extract, methanol and dichloromethane extract, chloroform and aqueous extracts, protein extract, and acetone extract. 2; ganoderic acid A, ganoderic acid B, ganoderic acid C1, ganoderic acid C2, ganoderic acid β, ganoderic acid S, ganoderic acid Sz, ganoderic acid H, ganoderic acid K, ganoderic acid TR, ganoderol A, ganoderol B, ganodermanondiol, ganodermanontriol. 3. Chloroform extract, aqueous extract, methanol extract, ethanol extract, acetone extract, and isolated ganoderin protein.

The most common oral fungus, *C. albicans*, is directly linked with candida oral infections. Several antifungal drugs have been used for many years, and a search for more agents, particularly from natural sources, that could exhibit anti-candidal properties is needed. *G. lucidum* containing toothpaste of different concentrations was evaluated in an *in vitro* study for its antifungal properties against *C. albicans*. *Ganoderma* containing toothpaste activity was evaluated against *C. albicans* in various ranges of minimum inhibitory concentration (MIC). The toothpaste showed antifungal activity against the tested microbes. Furthermore, many other fungal species of *Penicillium* and *Aspergillus* responded to the *G. lucidum* methanolic extract with remarkable zones of inhibition [138]. Different extracts of *G. lucidum* with varying compositions of organic solvents lead to accommodating results in terms of antifungal potential. Polyphenols in *G. lucidum* and other metabolites are known as the best assets of this variant for having an antifungal outcome. Phenolic components, cinnamic acid, *p*-hydroxybenzoic acid, and polysaccharides contribute to major *in vivo* and *in vitro* studies, with special reference to nine species of different genera [139]. *In vivo* and other extensive pharmacological research shows the reliability of *Ganoderma* species for use in various microorganisms and other parasitic diseases [140]. Extracted components in aqueous media possess a lesser potential to show the minimum zone of inhibition in comparison to alcoholic extracts. Mycelium and fruiting bodies of *G. lucidum* constitute the different activity patterns against different fungal strains, i.e., *Aspergillus, Bacillus, Candida, Acrimonium,* etc. One recent study supports the maximum inhibition against *C. albicans* in the ethanolic extract of *G. lucidum*, which later recorded the inhibition for other pathogenic fungal strains such as *T. rubrum*, *M. canis*, *A. niger*, *P. marneffei,* and *C. neoformans* in decreasing orders of effectiveness [141]. *G. lucidum* potential antimicrobial properties has been depicted in Fig. 4.

Mycelium of *G. lucidum* BCCM 31549 has been a significant source of glucan sulfate (GS), possessing therapeutic activities. GS have been evaluated for their antifungal demelanizing properties and nitrite oxide production from stimulated RAW264.7 macrophages [142]. GS exhibited antifungal activity against *Aspergillus niger* A60 with a minimum inhibitory concentration of 60 mg/ml and a minimum fungicidal concentration of 100 mg/ml. Possible applications of GS as a pharmacological, medicinal, and functional food ingredient with multifunctional benefits make it a potential agent against antimicrobial agents [143]. The antifungal effectiveness of *G. lucidum* extracts on different species of fungus can be seen in Table 4.

**Table 4:** *Ganoderma lucidum* antifungal properties against different species of *Aspergillus* using different solvent extracts.

Different *G. lucidum* extract	Different fungal species	MIC (μg/ml)	References
Chloroform Extract	*Aspergillus terreus*	450	[50] [50] [50] [50]
	*Aspergillus niger*	400	
	*Penicillium* sp.	400	
	*Aspergillus sydowii*	350	
Aqueous Extract	*Aspergillus sydowii*	200	
	*Aspergillus terreus*	200	
	*Aspergillus niger*	150	
	*Penicillium* sp.	150	
Methanol extract	*Aspergillus terreus*	300	
	*Aspergillus sydowii*	250	
	*Penicillium* sp.	250	
	*Aspergillus niger*	150	
Ethanol extract	*Penicillium* sp.	350	
	*Aspergillus terreus*	300	
	*Aspergillus sydowii*	200	
	*Aspergillus niger*	150	

## 4 Antioxidant effects

Oxidation is a biological process that is necessary for the creation of energy in many different types of living organisms. On the other hand, the uncontrolled generation of oxygen-derived free radicals is harmful to cells. Additionally, it has the potential to set off a chain reaction that will result in the production of additional free radicals. Free radicals lead to the interference and manipulation of proteins, damaging the genetic material as well as causing free radical-induced diseases and aging. Numerous synthetic antioxidants are currently being utilized on a widespread scale in an effort to lessen the harmful effects of oxidation on humans. On the other hand, findings from more recent studies suggest that synthetic antioxidants should be regulated because of the possible risks to human health, including liver damage and carcinogenesis [144,145]. Therefore, it is absolutely necessary to discover and make use of powerful naturally occurring antioxidants in order to shield the human body from the damage caused by free radicals and to lower the risk of an extensive range of diseases, including cancer, arthritis, and cardiac disorders [146,147].

**Fig. 5:**
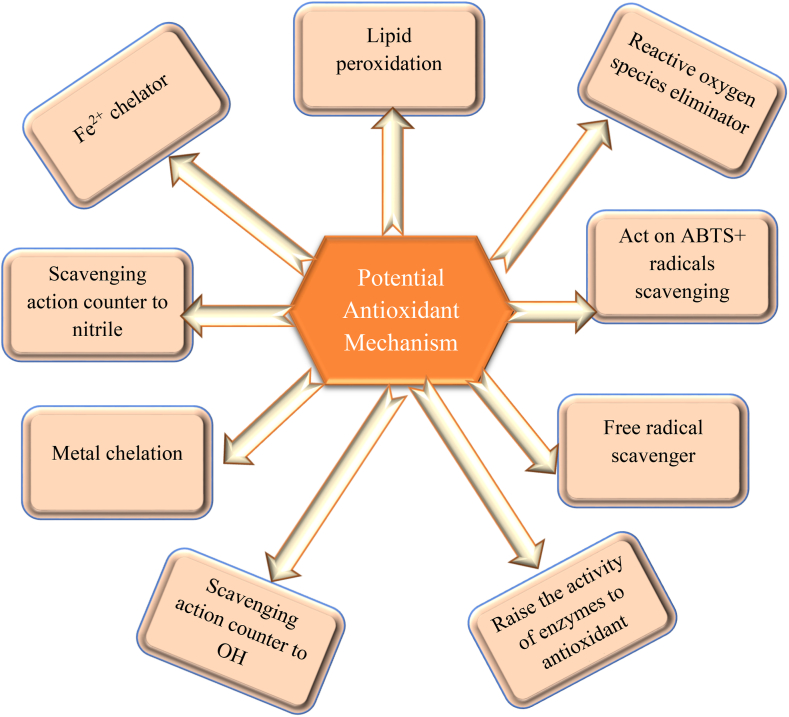
Diverse antioxidant effects produce by different constituents of mushroom.

*G. lucidum* isolated polysaccharides exhibit some potential anti-oxidant activities. It defends tissues contrary to reactive oxygen species (ROS)-induced toxicity as well as aids in maintaining the oxidative status of the body. Polysaccharides isolated from *G. lucidum* spores have been approved as drug based polysaccharides [148,149]. It has been shown that natural polysaccharides reveal a significant function as scavengers of free radicals in averting oxidative destruction in living organisms [150,151]. These natural polysaccharides have the potential to be studied as unique and significant antioxidants; earlier research suggested that polysaccharide antioxidant properties demonstrate the ability to boost the action of antioxidant enzymes, scavenge free radicals, reduce lipid peroxidation, and protect from free radical associated health hazards [152]. It is considered other than hormonal remedies applied in refractory myopathy treatment and in glucocorticoids combination therapy [153]. *In vivo* experiments have shown that *G. lucidum* polysaccharides exhibit anti-inflammatory and defensive properties in contrast to oxidative stress, particularly in liver, heart, skeletal muscles, and spleen diseases [154]. Polysaccharide antioxidant potency can be influenced by a wide variety of factors, such as its chemical constituents, structure, molecular mass, glycosidic linkage, and even mycelium culture conditions. Among all, molecular weight is the most essential structural property of polysaccharides. Polysaccharides with a lower molecular weight would have correspondingly stronger antioxidant activity [155,156]. *G. lucidum* structural analysis confirms *G. lucidum* polysaccharides (GL-PSs) are heteropolymers in which glucose is the main sugar component, whereas xylose, galactose, fucose, and mannose are present to lesser extents and in diverse conformations that include 1–3, 1–4, and 1–6-linkage β and α-D (or L)-substitutions [20,48]. It has been reported in a study that a low-molecular-weight glucan called β-1,3-glucan obtained from *G. lucidum* significantly increased the viability from 40 % to 80 % in H2O2-induced oxidative stress leukemia monocyte macrophage cell lines and reduced the formation of reactive oxygen species. Additionally, it was able to inhibit the activities of both acidic and neutral sphingomyelinases [157]. The ability of a homopolysaccharide based on mannose to boost the activity of antioxidant enzymes has also been demonstrated. Few studies have revealed that free radical scavenging properties are higher in conjugated forms of polysaccharides, for instance, polyphenolic-associated polysaccharides, polysaccharide-protein complexes, metal ion-enriched polysaccharides, and polysaccharide mixtures [158]. Various antioxidant properties of *G. lucidum* polysaccharides can be seen in Table 5. Different mechanisms are depicted in Fig. 5. The *Ganoderma* genus has lots of different species, including *Ganoderma tsugae*, *Ganoderma neo-japonicum*, and *Ganoderma atrum*, which also exhibit antioxidant properties. It can be seen in Table 6.

**Table 5:** Various antioxidant activities of polysaccharides obtained from *G. lucidum*.

Polysaccharides	Source	Activities	References
*Ganoderma lucidum* polysaccharides	Spore	• Decrease action of nitric oxide synthase, Cyt P450, myeloperoxidase and xanthine oxidase. • Decline levels of ROS in endothelial cells.	[159]
*Ganoderma lucidum* polysaccharides	Fruiting body	• Induce synthesis of glutathione peroxidase, catalase, glutathione S-transferase, mitochondrial succinate and dehydrogenase. • Reduce hyperlipidemia	[160]
*Ganoderma lucidum* polysaccharides	Fruiting body	• Induce synthesis of superoxide dismutase and catalase • Decrease lipid peroxidation and inflammatory cytokine IL-6, IL-1β and TNF-α	[161]
*Ganoderma lucidum* polysaccharides	Fruiting body	• Anti-superoxide radical ability and decline in lipid peroxidation. • Enhance the action of superoxide dismutase and catalase.	[48]
α-glucan and β-glucan	Fruiting body mycelium	• Raise IL-2, TNF-α and IFN-γ in human PBMC.	[162]
*Ganoderma lucidum* polysaccharides	Fruiting body	• Substantial anti-hydroxyl free radical activity. • Recover superoxide dismutase action. • Improve the insulin resistance.	[163]
*Ganoderma lucidum* polysaccharides	Fruiting body	• Reduction in IL-6 and IL-β, triglycerides and total cholesterol. • Rise glutathione peroxidase, catalase and superoxide dismutase • Reduce gut microbiota dysbiosis.	[164]

**Table 6:** Different species of *Ganoderma* having antioxidant properties with different mechanism of actions.

Different species of *Ganoderma*	Source	Potential action	Mechanisms	References
*Ganoderma lucidum*	Fruiting body	Antioxidant	• Enhance the production of superoxide dismutase, NADPH, manganese superoxide dismutase, CAT, and GSH • Protect the mitochondria in macrophages against induced injury	[[165], [166], [167]]
*Ganoderma neo-japonicum*	Fruiting body	Neuroprotective	• Encourage neurogenesis via MEK/ERK1/2 and PI3K/Akt signaling pathways	[168]
*Ganoderma antler*	Fruiting body	Antioxidant	• Scavenging free radicals and decreasing oxidative stress	[168,169]
*Ganoderma capense*	Culturing mycelium powder	Antioxidant	• Hydroxyl radical-scavenging abilities	[170]
*Ganoderma atrum*	Fruiting body	Immunomodulation	• Encourage the production of IL-2 and enhance the activation of spleen lymphocytes • Induce the release of TNF-α during macrophage activation	[171,172]

## 5 Development of secondary metabolites

**Fig. 6:**
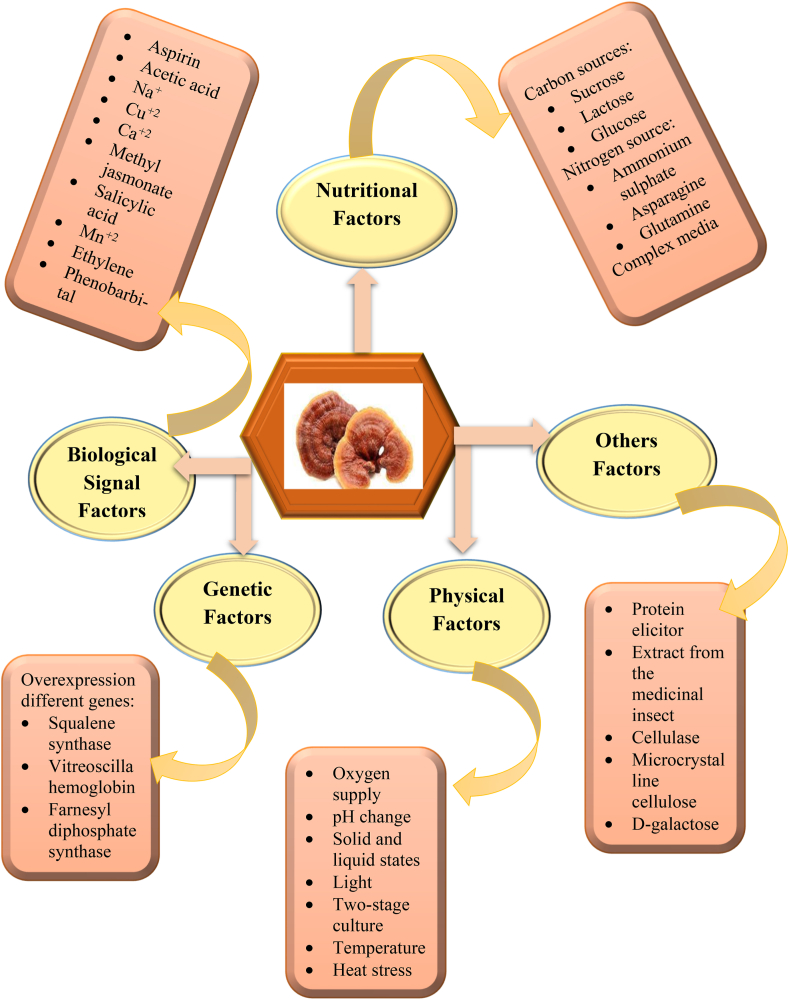
Various factors including nutritional, physical, biochemical and genetic that potentiate the yield of secondary metabolites production.

The demand for its fruiting bodies and/or mycelium biomass in international markets is growing repeatedly. This demand prompted the development of various methods rather than traditional methods for the production of *G. lucidum* and its secondary metabolites to meet the demand. People have been using various methods of cultivation, such as bags filled with wood or straw and wood logs, for decades. For small and pilot plant production, biotechnology has been used for cultivation in bioreactors on solid substrates or with liquid substrates as submerged fermentation [[173], [174], [175]]. Recent research has paid a lot of attention to improving the production of secondary metabolites [176]. According to the most recent research, there are physical, genetic, biochemical, and nutritional factors that affect the biosynthesis of *G. lucidum* secondary metabolites [177]. These factors potentiate the production of secondary metabolites, particularly ganoderic acids (Fig. 6). Most studies have been done on the terpenoids of *G. lucidum*, owing to their pharmacological and nutritional potential outcomes in different diseases and the abundance of their presence in this mushroom [1,178]. There are several factors that improve secondary metabolite production, among them signal transduction, which makes a significant contribution to the biosynthesis of GAs. Na+, Ca2+, reactive oxygen species, and cyclic adenosine monophosphate play important roles in the signaling and regulation of ganoderic acids biosynthesis [179]. Ca^2+^ controls a wide range of physiological changes, cellular processes, and secondary metabolism [180]. It has reported that variation of calcium intracellularly activates the receptors and regulates the downstream genes [181,182]. When calcium ions are added to static liquid cultures, the ganoderic acids production goes up. While heat stress increases the amount of Ca^2+^ in the cytosol, that leads to improve the biosynthesis (Zhang et al., 2016). While other metal ions like Cu^+2^ and Na ^+^ also play a significant role in the better production of ganoderic acids. Genetic factors are also a substantial approach to getting the bioactive constituents of different *Ganoderma* species to make bioactive products, especially secondary metabolites [182].

There are several new methods that have been studied and work well to increase the production of secondary metabolites, such as media, temperature, light, and pH. Oxygen deprivation is an encouraging source for secondary metabolite production. It has been seen that GA-S, GA-T, GA-Me, and GA-Mk production are upraised in hypoxia-induced mycelia. Triterpene biosynthesis enzymes such as 3-hydroxy-3methylglutaryl coenzyme A reductase, mevalonate-5 pyrophosphate decarboxylase, and squalene synthase gene expression were also enhanced in hypoxia compared to high oxygen atmospheres. Manipulating the mechanistic approaches also helps to increase secondary metabolites (particularly GAs and polysaccharide biosynthesis) by changing the pH and culture. Production of GAs has been reported to be higher at pH 5.5 and 6.5 [183,184]. Furthermore, the production of antimicrobial secondary metabolites from *G. lucidum* can be improved by using different food sources, for instance, carbon and nitrogen or a combination of both carbon and nitrogen sources. It has been found that the production of GAs like GA-Me is affected by the amount of carbon [185]. While several nitrogen sources, such as asparagine, glycine, glutamine, and ammonium sulfate were evaluated to check their limiting factors for the production of different secondary metabolites [[185], [186], [187]].

## 6 Recent advances in genetic and metabolic engineering

Genetic engineering is the process of altering an organism's genetic makeup in order to improve or add novel characteristics. The goal of metabolic engineering is to enhance the synthesis of desirable substances and secondary metabolites by modifying an organism's metabolic pathways [188]. These two methods have been investigated to improve *G. lucidum* medicinal properties and yield. Researchers have concentrated on boosting the production of bioactive substances in *G. lucidum*, which are responsible for its medicinal properties. These substances include triterpenoids, polysaccharides, and ganoderic acids [189,190]. This is accomplished by either importing genes from other organisms that encode enzymes with desirable activity or by overexpressing important genes involved in the synthesis of these compounds. The complete genome sequencing of *G. lucidum* has provided valuable insights into its genetic makeup and metabolic pathways [191]. An advanced method for enhancing the production of GAs is the use of genetic engineering, such as squalene synthase (SQS) gene expression [183]. According to reports, overexpression of SQS increases the synthesis of GA-S, GA-Me, GA-T, and GA-MK to varying degrees [192]. By introducing vitreoscilla hemoglobin (VHb) gene expression in *G. lucidum,* the synthesis of GAs (GA-S, GA-T, GA-Mk, and GA-Me) was improved relative to the wild types strain [190,193]. Additionally, a recent study indicates that overexpressing the homologous farnesyl diphosphate synthase (FPS) gene in *G. lucidium* was a successful strategy for increasing secondary metabolite production. They found that in the transgenic strain, overexpression of the FPS gene increased the level of GAs, including GA-T, GA-S, and GA-Me. Furthermore, a study on mutated *sdhB*, encoding the iron-sulfur protein subunit of succinate dehydrogenase, was used as a selection marker in a homologous genetic transformation system for *G. lucidum*. *Agrobacterium tumefaciens*-mediated transformation technique was used to overexpress a truncated *G. lucidum* gene expressing the catalytic domain of 3-hydroxy-3-methylglutaryl coenzyme A reductase (HMGR). The HMGR gene was overexpressed, resulting in a two-fold rise in ganoderic acid production. It also boosted intermediate concentrations (squalene and lanosterol) and the activation of downstream genes such as squalene synthase, farnesyl pyrophosphate synthase, and lanosterol synthase. The transgenic basidiomycete *G. lucidum* is a promising system for metabolic engineering to produce higher secondary metabolites [194]. New developments in genetic and metabolic engineering are opening the door to the creation of *G. lucidum* strains with improved production traits and increased therapeutic potential. To fully realize the therapeutic potential of *G. lucidum*, more research is necessary in the field of genetic and metabolic engineering.

## 7 Future scenarios of secondary metabolites as antioxidants and antimicrobial agents

There are several potential future developments for *G. lucidum* as an antioxidant that can be included in functional foods and supplements. Extracts are already used in some functional foods and dietary supplements due to their antioxidant and immune-modulatory properties [53]. As more research is conducted on the health benefits of *G. lucidum* as an antioxidant, demand for functional foods and supplements containing this mushroom is increasing across the world. The development of new skincare as an antioxidant is important for protecting skin from damage caused by free radicals, which can contribute to premature aging and other skin disorders. *G. lucidum* extracts have been shown to possess antioxidant activity against several skin pathogens, making them a potential ingredient in skincare products such as cosmetic base creams [195]. Antioxidants are important for protecting the environment from damage caused by pollutants and other environmental stressors. *G. lucidum* has been shown to have potential for bioremediation as it can break down toxins and pollutants in soil and water. This suggests that it could be used to protect the environment from oxidative stress caused by pollutants. Overall, the antioxidant properties of *G. lucidum* suggest that it has promising future prospects as a natural source of antioxidants, with potential applications in medicine, skincare, environmental protection, and disease prevention [195,196]. However, further research is needed to fully understand the mode of action and various applications. *G. lucidum* has been shown to possess potent antimicrobial activity against a variety of bacteria, viruses, and fungi [92]. There are several potential future developments for *G. lucidum* as an antimicrobial agent that could be possible, such as the development of new antibiotics, natural preservatives, bioremediation, and personal care products [2,197]. Overall, the antimicrobial properties of *G. lucidum* suggest that it has promising future prospects as a natural source of antimicrobial agents. However, further research is needed to fully understand the mechanisms of action and potential side effects of *G. lucidum* extracts and to optimize their production and formulation for different applications.

## 8 Side effects

Despite potential health benefits, there is controversy surrounding the use of *G. lucidum* as a medicinal supplement. Some studies have shown that it may have potential side effects, such as liver toxicity and allergic reactions [198]. *G. lucidum* may cause allergic reactions in certain people. Mild symptoms like skin rashes are examples of mild allergic responses. Some people have reported temporary symptoms of fatigue, thirst, bloating, abnormal sweating, frequent urination, and diarrhea after taking *G. lucidum* powder extract [[199], [200], [201]]. *G. lucidum* has been reported to have mild blood-thinning properties. While this can be valuable for certain individuals, such as those with a risk of blood clots and those using blood-thinning drugs like aspirin and warfarin, *G. lucidum* in combination with these medications may enhance the bleeding risk or interfere with the effectiveness of the medication [200]. *G. lucidum* has been found to have a hypotensive effect. While this can be beneficial for people with hypertension, it may cause complications for those with already low blood pressure or people taking drugs to lower blood pressure [202]. There is also concern about the quality and purity of *G. lucidum* supplements, as many products on the market may contain harmful contaminants or low levels of the active ingredients.

## 9 Concluding remarks

It has been shown that *G. lucidum* has a lot of different bioactive components that act as potential sources for health-promoting agents. So far, most studies have been done on groups of compounds such as triterpenoids and polysaccharides. The structural variability of the obtained biologically active compounds makes this mushroom exceptional among the other mushrooms as a health-promoting agent used to prevent and treat a wide range of diseases. In this review, the antimicrobial effects and antioxidant activities of *G. lucidum* isolated compounds and extracts have been focused on. Demand for this mushroom is rising across the world as a medicinal, nutraceutical, and functional food. The lack of standardized extracts in clinical studies is a problem that makes it hard to develop different antimicrobial and antioxidant agents. Additional investigation is essential, with a focus on the definite amount of standardized extracts of pharmacologically active compounds. *In vivo* and *in vitro* research should be done on these characterized extracts to find out the exact amount of a certain compound that could be used in advanced clinical and experimental studies. Furthermore, studies and more research could help make it easier to create medicinal and nutraceutical formulations that could be used to treat a wide range of diseases, particularly those caused by viruses, fungi, and bacteria. The biologically active substances must also be validated with regard to their side effects and toxicity to demonstrate their safety and effectiveness. Moreover, research and trials are being conducted to determine the efficacy of numerous compounds in support of the antimicrobial activity and antioxidant properties obtained from *G. lucidum.*

## CRediT authorship contribution statement

**Md Faruque Ahmad:** Methodology, Data curation, Conceptualization. **Abdulrahman A. Alsayegh:** Supervision. **Fakhruddin Ali Ahmad:** Resources, Formal analysis. **Md Sayeed Akhtar:** Writing – review & editing, Formal analysis. **Sirajudeen S. Alavudeen:** Resources. **Farkad Bantun:** Visualization. **Shadma Wahab:** Methodology. **Awais Ahmed:** Writing – review & editing. **M. Ali:** Writing – review & editing. **Ehab Y. Elbendary:** Data curation. **António Raposo:** Investigation. **Nahla Kambal:** Formal analysis. **Mohamed H. Abdelrahman:** Visualization.

## Declaration of competing interest

The authors declare that they have no known competing financial interests or personal relationships that could have appeared to influence the work reported in this paper.
